# The efficiency and safety of tranexamic acid for reducing blood loss in open myomectomy: A meta-analysis of randomized controlled trials: Retraction

**DOI:** 10.1097/MD.0000000000009793

**Published:** 2018-01-26

The article, “The efficiency and safety of tranexamic acid for reducing blood loss in open myomectomy, A meta-analysis of randomized controlled trials”,^[1]^ which appeared in Volume 96, Issue 23 of *Medicine*, is being retracted for errors in the data collection. The errors affected the results and conclusion of the paper.
